# Electrical and Hormonal Biomarkers in Cachectic Elderly Women with Chronic Heart Failure

**DOI:** 10.3390/jcm9041021

**Published:** 2020-04-04

**Authors:** Grzegorz Sobieszek, Tomasz Powrózek, Marcin Mazurek, Anna Skwarek-Dziekanowska, Teresa Małecka-Massalska

**Affiliations:** 1 Department of Cardiology, 1st Military Clinical Hospital with the Outpatient Clinic, 20-048 Lublin, Poland; anetask@gmail.com; 2 Department of Human Physiology, Medical University of Lublin, 20-080 Lublin, Poland; marcinmazurek1212@gmail.com (M.M.); t.malecka@gmail.com (T.M.-M.)

**Keywords:** chronic heart failure, cachexia, irisin, bioimpedance analysis, biomarker

## Abstract

Background: Cachexia is an unfavorable metabolic syndrome causing involuntary weight loss followed by muscle wasting, which can lead to the exacerbation of chronic heart failure (CHF), and considerably increases mortality rate among CHF patients. Unfortunately, until now it has not been possible to determine factors that could improve clinical options for cachexia management or enable the identification of patients at risk of its development. We assessed how cachexia conditions in CHF reflect cardiac and laboratory parameters in comparison with non-cachectic patients. Methods: 66 women were enrolled to the study group and underwent meticulous screening, according to recent clinical guidelines, in order to enable CHF and cachexia detection. Body composition was evaluated by bioelectrical impedance analysis (BIA) and laboratory tests were supplemented by analysis of plasma circulating irisin. Results: A negative correlation between irisin concentration and both CRP and TNF-α was recorded (*R* = −0.362 and *R* = −0.243; *p* < 0.05). Irisin concentration positively correlated with EF% (*R* = 0.253; *p* = 0.046) and negatively with LVESd, LVEDd and NT-proBNP (*R* = −0.326, −0.272, and −0.320; *p* < 0.05). Both low levels of circulating irisin and Capacitance of membrane (Cm) were selected as unfavorable factors affecting cachexia in CHF patients (OR = 1.39 and 34.49; *p* < 0.05). Combination of Cm, irisin, CRP and albumin demonstrated sensitivity of 93.3% and specificity of 85.3% (AUC = 0.949) for distinguishing between cachectic and non-cachectic CHF patients. Conclusions: Selected parameters reliably reflect cachectic conditions in CHF, and the proposed approach for cachexia based on the combined analysis of at least a few non-invasive markers could offer new opportunities for improving clinical outcomes in CHF patients.

## 1. Introduction

Chronic heart failure (CHF) is a disease with growing incidence in adults in highly developed countries, and itsrisk factors include presence of other cardiovascular diseases or the coexistence of chronic diseases [1,2]. Due to metabolic alterations associated with the disease, CHF patients are increasingly diagnosed with heart failure-induced cachexia (cardiac cachexia; CC), which is diagnosed in 8–42% CHF patients, and the mortality rate is roughly 20–30%. Progression of cachexia in CHF patients is based on the reduced myocardial perfusion, cardiomyocyte decline and the depletion of high energy stores (reduced cardiac function and increased catabolic state of the body) [3,4,5]. Until now, there is lack of literature reports concerning combined use of hormonal markers (e.g., irisin) and other tools (bioelectrical impedance analysis, BIA) in the management of cachexia in patients with CHF.

Irisin, a thermogenic adipomyokine, regulates body energy metabolism and then its conversion in the form of ATP, but also participates in the browning of adipose tissue [6,7]. The blood concentration of irisin is significantly altered in patients suffering from acute coronary syndrome, especially in myocardial infarction (AMI), ST-Elevation Myocardial Infarction (STEMI) and stable coronary disease. Irisin seems to be a clinically promising marker reflecting cardiovascular system efficiency and the metabolic condition of the entire body of patients with CHF [8,9]. BIA as a non-invasive method reliably evaluates body composition and its nutritional status at the cellular level. The two potentially clinically useful parameters, which can be obtained from BIA, are membrane capacitance (Cm) and phase angle (PA). They show clinical usefulness in the body composition assessment and the detection of malnutrition and demonstrate a prognostic value in various diseases accompanied by malnutrition, including CHF [1,10,11,12].

The aim of the study was to assess how cachexia conditions in CHF females reflects cardiac and laboratory parameters in comparison with non-cachectic patients. Our study is limited only to women, in order to be able to interpret the electrical, hormonal and biochemical changes. Age, sex and body mass index are the determinants of the electrical parameters.

## 2. Patients and Methods

### 2.1. Study Group

Sixty-six women (mean age: 77 ± 9 years) with newly diagnosed CHF were enrolled to the study group. All study participants were inpatients diagnosed and treated at the Clinic of Cardiology and Internal Medicine, Department of Cardiology, Military Hospital in Lublin, Poland between 2017 and 2019. CHF was diagnosed according to the recent European Society of Cardiology (ECS) criteria, that are based on a patient’s clinical evaluation and echocardiographic assessment supplemented by blood examination, including a serum concentration of N-terminal prohormone of brain natriuretic peptide (NT-proBNP), lipid profile, creatinine and hemoglobin level [13]. The extent of the disease was classified according to New York Association (NYHA) guidelines, which qualify patients to four functional classes: I–IV based on the symptom’s severity [14]. For the study protocol, the defined inclusion and exclusion criteria were applied. The inclusion criteria to the study were as follows: (1) age >18 years, female gender and Polish ethnicity; (2) signed consent to participate in the study protocol; (3) newly diagnosed CHF. The exclusion criteria were as follows: (1) extreme renal failure or eGFR<15 mL/min/1,73m^2^; (2) acute coronary syndrome; (3) a coronary artery bypass grafting within the last 6 months; (4) hyper- or hypothyroidism; (5) presence of a heart pacemaker, cardioverter or defibrillator, as well as a lack of metallic implants. Detailed baseline characteristics of the study group is presented in Table 1.

Study protocol was approved by Bioethical Commission in Medical University of Lublin (no of consent: KE-0254/64/2017). Prior to the study, all patients signed informed consent forms.

### 2.2. Nutritional Assessment and Cachexia Detection

Cachexia was diagnosed according to the criteria proposed by Evans WJ et al: a weight loss of at least 5% or more in 12months or less in the presence of underlying illness, plus three of the following criteria: decreased muscle strength, fatigue, anorexia, low fat-free mass index, abnormal biochemistry (increased inflammatory markers: C-reactive protein >5.0 mg/L), IL-6 >4.0 pg/mL), anemia (<12 g/dL) and low level of serum albumin (<3.2 g/dL)) [15].

### 2.3. Bioelectrical Impedance Analysis

BIA measurement was conducted with the use of an ImpediMed bioimpedance analysis SFB7 BioImp v1.55 device (Pinkenba, Queensland, Australia). To provide reliable measurements, we applied similar BIA conditions for all study participants—the data was collected in the morning and on an empty stomach. Prior to examination, patients were lying in a bed in the supine position (lying on the back and their legs and arms were not in contact with the torso) for at least five minutes, to equalize a body fluid level. All measurements were performed on the right side of the body (right-side location of the electrodes). Cm, Pa, Z5 and Z200 values were automatically obtained from the BIA equipment. Then the Z200/Z5 ratio was calculated. The following parameters reflecting body composition of the patients were derived from the BIA: fat mass (FM) and fat-free mass (FFM).

### 2.4. Plasma IrisinConcentration

From each study participant, 5 mL of whole blood was collected in order to assess plasma irisin level. After plasma separation, samples were stored at −80 °C until the analysis. Plasma irisin level was measured according to the manufacturer’s protocol, with the use of the dedicated Irisin ELISA Kit (BioVendor, Brno, Czech Republic). The detection range of the kit was 0.001–5 µg/mL, and the sensitivity was equal to the minimal detectable dose of this kit (<1 ng/mL).

### 2.5. Statistical Analysis

Statistical analysis was conducted with the use of MedCalc computer software (version 15.3) (MedCalc, Ostend, Belgium). All graphs illustrating results of the statistical analysis were generated by the same applied statistical software. Data distribution of the collected variables was checked by the Shapiro-Wilk test. Based on the result of data distribution analysis, either parametric student’s *t*-test or non-parametric Mann-Whitney *U*-test were applied to compare anthropometric, biochemical, nutritional and cardiac parameters between the cachectic and non-cachectic group of HF patients. Correlation between irisin and studied parameters was tested with the use of Spearman’s rank correlation. The uni- and multivariate logistic regression model with the odds ratio calculation (OR) was applied to select factors affecting the cachexia incidence in female CHF individuals. Receiver operating curves (ROC) with area under the curve (AUC) calculation were used to assess accuracy of the selected parameters for distinguishing between cachectic and non-cachectic CHF patients. Results with *p* values below 0.05 were considered as being statistically significant.

## 3. Results

Median concentration of plasma irisin was significantly decreased in cachectic women compared with non-cachectic ones (median irisin concentration: 7.12 µg/mL (IQR: 5.94–9.42) and 7.61 µg/mL (IQR: 5.29–10.39); *p* = 0.022). Moreover, increased level of inflammatory markers was noted in cachectic patients compared to women without symptoms of cachexia (median CRP level: 10.95 mg/L (1.96–26.20) and 3.10 mg/L (1.45–4.95); *p* = 0.005; median TNF-α level: 4.48 pg/mL (3.49–5.13) and 3.29 pg/mL (3.07–4.91); *p* = 0.032). Regarding parameters reflecting cardiac function, we recorded significant differences between analyzed groups. First of all, cachectic patients demonstrated reduced EF% (mean: 42 ± 13% and 48 ± 9%; *p* = 0.039) and a significantly higher level of NT-proBNP (median: 3476 pg/mL (IQR:1690–5773) and 1176 pg/mL (IQR:716–2605); *p* < 0.001). Severe cardiac symptoms defined as NYHA III and IV class were more frequent among cachectic patients. Some 61.8% of women belonging to cachectic group were qualified as either NYHA III or IV class, while the aforementioned proportion was 34.4% in non-cachectic patients (*p* = 0.030). All of the following parameters reflecting the nutritional status of CHF patients—body weight, BMI, FM, FFM and albumin—were significantly reduced in the cachexia group (*p* < 0.05). Also, the significant differences in BIA parameters reflecting the nutritional status of the body cells were observed among patients. The most significant difference between cachectic and non-cachectic patients was recorded for the Cm (median Cm: 0.860 nF (IQR: 0.670–1.070) and 1.280 nF (IQR: 0.916–1.789); *p* < 0.001). Differences in studied parameters between cachectic and non-cachectic patients are summarized in Table 2.

In the subsequent part of the study, we investigated correlation between concentration of plasma irisin in CHF patients, and the studied anthropometric, nutritional and cardiac parameters. The statistically significant results of the correlation study are presented in Table 3. 

**Figure 1:**
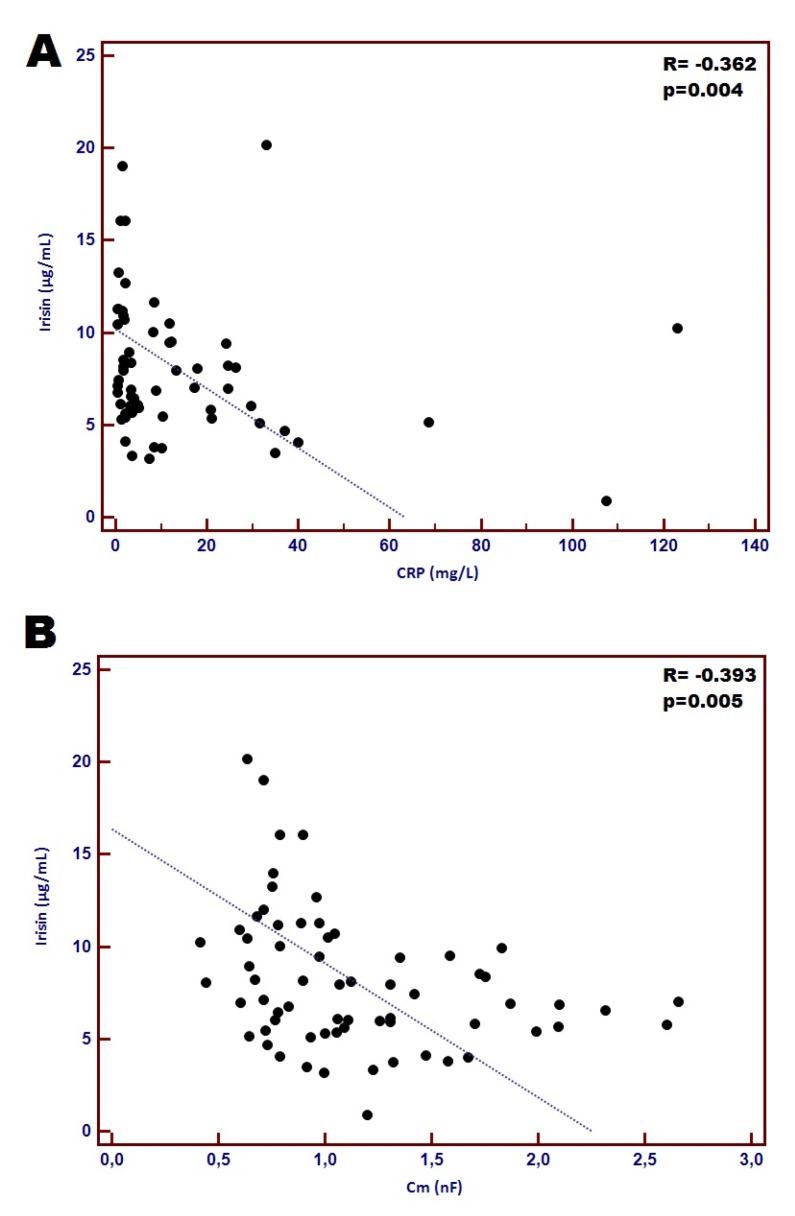
Correlation between concentration of plasma irisin and CRP (**A**) and correlation between plasma irisin and Cm (**B**).

In the whole studied group concentration of plasma irisin significantly correlated with selected parameters reflecting inflammatory, nutritional and cardiac status. We found negative correlation between the level of CRP and TNF-α and irisin; CHF patients with higher irisin concentration had reduced inflammatory response represented by CRP and TNF-α (*R* = −0.362 and *R* = −0.243; *p* = 0.004 and *p* = 0.044, respectively). Correlation between CRP and irisin is shown in Figure 1A. Regarding cardiac parameters, irisin concentration positively correlated with EF% (*R* = 0.253; *p* = 0.046) and negatively with LVESd, LVEDd and NT-proBNP (*R*= −0.326, −0.272, and −0.320; *p* = 0.009, 0.030, and 0.010, respectively). Moreover, patients with lower irisin concentration had higher Cm values (*R* = −0.393; *p* = 0.005) (Figure 1B).

Using logistic regression analysis (uni- and multivariate), the factors significantly affecting the chance of cachexia incidence in CHF patients were identified (Table 4). The univariate analysis revealed albumin concentration and Cm value as the factors, that most significantly affect probability of cachexia in CHF patients (OR = 33.18 and OR = 10.76, respectively). Similarly, the multivariate analysis model indicates that albumin and Cm emerge as the independent factors related to cachexia incidence in CHF women (OR = 50.48 and OR = 34.49, respectively).

**Figure 2:**
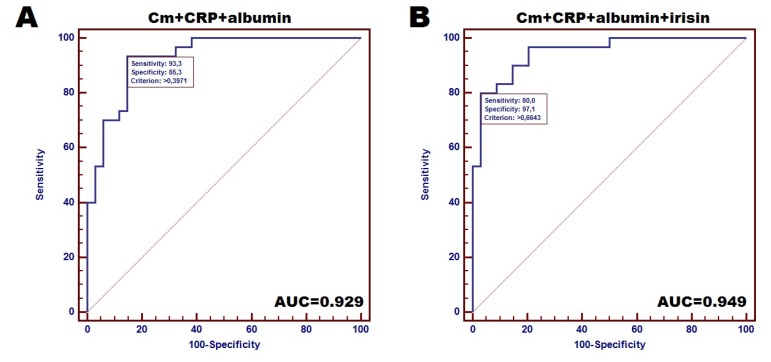
Accuracy of designed tests for distinguishing between cachectic and non-cachectic CHF female patients: (**A**)—Receiver operating curves (ROC) for combined analysis of 3 markers (Cm, CRP and albumin), (**B**)—ROC for 4 markers-based tests (combination of CM, CRP, albumin and irisin).

Finally, we also estimated diagnostic accuracy of irisin, inflammatory markers and Cm for distinguishing between cachectic and non-cachectic CHF patients. Among the studied factors, the strongest individual diagnostic accuracy reflected by AUC was achieved by Cm (AUC = 0.787; sensitivity and specificity: 52.9% and 90.6%, respectively). Notably, we observed improvement of the diagnostic power of the designed test combining at least two markers. The highest accuracy was demonstrated by test based on the combination of Cm with albumin (AUC = 0.917). Combined analysis of Cm, CRP and albumin demonstrated especially high diagnostic sensitivity of 93.3% with a specificity of 85.3% (AUC = 0.929) (Figure 2A), however, the combination of Cm, CRP, albumin and irisin achieved a reduction in the sensitivity to 80%, but the considerable specificity of 97.1% was demonstrated (AUC = 0.949) (Figure 2B) as distinguishing between cachectic and non-cachectic CHF patients. 

Diagnostic accuracy of particular markers is demonstrated in the Table 5. 

## 4. Discussion

According to the literature, there are no reports of studies combining the use of biochemical markers and BIA in the diagnosis of cachexia in patients with CHF. Based on the literature findings, laboratory parameters have indicated that irisin is a clinically interesting adipomyokine reflecting cardiovascular system efficiency. The reason why irisin was chosen for our research are briefly presented below.

El-Mottaleb NA et al. found irisin to be a useful biomarker in the diagnosis of myocardial infarction with or without heart failure [9]. The authors evaluated eighty-six subjects (33 patients with myocardial infarction, 33 patients with myocardial infarction and heart failure, and 20 controls) and found negative correlations between irisin and BMI, WHR, SBP, DBP, troponin-I, CK-MB, TNF-α, TC, TGs, and LDL-C. However, a positive association was observed between irisin and LVEF and HDL-C. Another study by Hsieh IC et al. showed that serum concentration of irisin might be a useful marker in STEMI monitoring. The level of this adipomyokineis elevated in post-STEMI patients with increased risk for adverse cardiovascular events. That is why the authors concluded that therapies targeting irisin may represent a new direction in future treatment [16]. A study by Silvestrini A et al. found higher level of irisin in HFpEF than in HFrEF patients (7.72 ± 0.76 vs. 2.77 ± 0.77 ng/mL, respectively). The authors correlated these findings with total antioxidant capacity (TAC), as an index of oxidative stress, and found an inverse correlation between irisin and TAC in HFpEF but not in HFrEF, which led them to conclude that different pathophysiological mechanisms are involved in the two CHF subtypes, and that oxidative stress modulates irisin secretion [8]. According to recent findings, circulating adropin is also an independent risk factor for heart disease, and its plasma level increased with the severity of HF. Interestingly, the study by Kalkan AK et al. evaluated adropin and irisin levels in cachectic and non-cachectic subjects, and the relationships between the levels of these proteins and clinical and laboratory parameters in patients with HFrEF. Cachectic patients (*n* = 44, mean age: 65.4 ± 11.2; 61.4% men) were identified in the study group of 86 patients. Serum irisin level was significantly higher in the cachexia group than in the controls (Irisin (µg/mL); 2.6 (IQR:2.2–4.4) vs. 2.1 (IQR:1.8–2.4); *p* = 0.001), and positively correlated with BNP levels and NYHA class, and negatively correlated with BMI and serum albumin level (all *p* values: <0.001) [5]. The results suggest that adropin and irisin may be novel markers of cardiac cachexia in patients with heart failure with a reduced ejection fraction.

It is worth mentioning that alterations in body composition, like the loss of skeletal muscle mass (sarcopenia or/and cachexia) or fat mass, are frequent in heart insufficiency. The prevalence and clinical consequences are very often underestimated. The assessment of cachexia among those patients has been a great challenge, as researchers use many different criteria of cachexia. Due to this fact, it has been difficult to make solid conclusions for further treatment strategies.

Our group was divided into cachectic and non-cachectic subjects, according to the criteria proposed by Evans WJ et al. These criteria include laboratory, clinical and functional parameters like: weight loss of at least 5% or more in 12months or less in the presence of underlying illness, plus three of the following criteria: decreased muscle strength, fatigue, anorexia, low fat-free mass index, abnormal biochemistry (increased level of inflammatory markers: C-reactive protein >5.0 mg/L), IL-6 >4.0 pg/mL), anaemia (<12 g/dL) and low concentration of serum albumin (<3.2 g/dL).

In our research, CHF female patients who suffered from cachexia exhibited a significantly poorer general condition reflected by BIA, cardiac and laboratory parameters, compared to non-cachectic individuals. Regarding cardiac parameters, 61.8% cachectic women and 34.4% non-cachectic females were qualified to NYHA III and IV class (*p* = 0.030). The cachectic patients demonstrated a significant reduction of cardiac performance measured by EF% (*p* = 0.039). 

According to recent findings, irisin is considered as an anti-inflammatory myokine against the pro-inflammatory activation of adipocytes, and is assumed to serve as a putative agent eliciting cardioprotection. Our results should comply with the above-mentioned observations. First, we noted significantly lower level of circulating irisin in cachectic CHF patients compared to non-cachectic study participants (median: 7.12 µg/mL vs. 7.61 µg/mL; *p* = 0.022). This result is different from that reported by Kalkan et al. [5]. The reason for this might be the group of patients (our group consisted only of women, whereas the a above-mentioned study included both women and men), different criteria for the cachexia assessment (in our research we used the ones proposed by Evans et al. [15] whereas in Kalkan study the ones applied by [17,18]). Second, the significant negative correlation between plasma irisin concentration and the level of both CRP and TNF-α was recorded (*R* = −0.362 and *R* = −0.243; *p* = 0.004 and *p* = 0.044, respectively). Perhaps this can explain the more favourable cardiac and nutritional condition of non-cachectic patients. They had reduced inflammatory response represented by CRP and TNF-α. This finding seems to be supported by the positive correlation between irisn concentration and EF%, as well as the negative correlation with NT-proBNP level (*R* = 0.253 and *R* = −0.320; *p* = 0.046 and *p* = 0.010, respectively). Moreover, patients with lower irisin concentration had higher Cm values (*R* = −0.393; *p* = 0.005), however, CHF patients suffering from cachexia had lower levels of Cm (*p* < 0.001). Interestingly, despite this non-pairability, both the low level of circulating irisin and Cm were selected as unfavourable factors affecting cachexia in CHF patients (OR = 1.39 and 34.49, respectively). We also estimated diagnostic accuracy of irisin and Cm for distinguishing between CHF patients with the presence of cachexia and non-cachectic individuals. Combination of Cm and irisin with CRP and albumin concentration demonstrated considerable diagnostic accuracy for distinguishing between cachectic and non-cachectic CHF patients (sensitivity of 93.3% and specificity of 85.3%; AUC = 0.949).

Among studies assessing the impact of wasting syndromes (cachexia and sarcopenia) in HF on functional parameters, it is worth mentioning one recently published by Emani et al. [19]. In a group of 207 ambulatory male patients with clinically stable CHF, cachexia was present in 39 (18.8%) of 207 patients, 14 of whom also fulfilled the characteristics of sarcopenia (sarcopenia + cachexia group, 6.7%). Patients with sarcopenia were weaker and had a lower exercise capacity than both the patients without wasting syndromes and cachectic subjects in the CHF group. Handgrip strength, quadriceps strength, peak oxygen uptake (VO2), distance in the 6-minute walk test (6MWT) and quality of life (QoL) results were the lowest in the sarcopenia + cachexia group vs. the no wasting syndrome group (*p* < 0.05 for all). This shows that loss of muscle with or without weight loss appears to have a pronounced influence on functional parameters like handgrip strength, quadriceps strength, VO2, 6MWT and QoL. Recently, a new class of biomarkers, circulating microRNA, is emerging, as they may provide additional pathophysiological information, helping to improve prognostic assessment. A number of circulating microRNAs are known to be altered in cachexia and sarcopenia [20,21]. Interestingly, among screened microRNAs, several are also candidate markers in heart failure [22,23], as well as some cachexia-related circulating microRNAs, such as microRNA-21 and microRNA-133, and are promising biomarkers in heart failure thatmight be able to help distinguish underlying etiology, including cachexia [24].

Unfortunately, our group was focused only on cachectic state without including sarcopenia impact, which is, of course, one of the limitations of the study. Additionally, the study includes only females, and this is another limitation of the research, which reflects the presence of a selection bias. However, our findings might be useful for clinical practice in distinguishing cachectic patients with CHF (by measuring Cm by BIA and laboratory parameters- irisin, albumin, CRP) in order to monitor these patients more accurately, as the diagnostic accuracy is very high (sensitivity of 93.3% and specificity of 85.3%; AUC = 0.929).

## 5. Conclusions

There are neither established criteria nor clinical guidelines allowing early detection and management of CC. However, both the high prevalence of cachexia among CHF individuals and serious conditions affected by this multifactorial syndrome, encourage facing this clinical problem with an interdisciplinary approach involving the analysis of different biomarkers. Our findings demonstrate novel perspectives for cachectic CHF patients, which include the following prospective benefits: selection of CC risk group, prediction of treatment outcomes and disease course. Our approach has not been practiced before, but we believe that combined analysis of at least a few non-invasive markers, such as irisin, Cm, albumin and inflammatory markers, could improve clinical opportunities for CC management. However, the putative clinical utility of the proposed diagnostic procedure needs to be confirmed by studies involving a larger number of enrolled cases.

## Figures and Tables

**Table 1:** Characteristics of the study group (ACEi—Angiotensin-converting-enzyme inhibitors; ARBs—angiotensin receptor blockers; BMI—body mass index; Cm—capacitance of membrane; EF—ejection fraction; FM—fat mass; FFM—fat-free mass; HRT—Hormone replacement therapy; LAD—left anterior descending artery; LVEDd—left ventricular end-diastolic diameter; LVESd—left ventricular end-systolic diameter; NYHA—New York Heart Association; Pa—phase angle (50kHz); PASP—pulmonary artery systolic pressure; RVOT—right ventricular outflow tract; SGA—subjective global assessment; TAPSE—tricuspid annular piane systolic excursion) ^†^—non-parametric Mann-Whitney *U*-test; the other parameters were tested by parametric student’s *t*-test.

Factor		Study Group (*n* = 66)
Age (years)		77 ± 9.0
Weight (kg)		77 ± 18.0
BMI (kg/m^2^)		29.75 ± 6.52
FM (kg)		26.45 ± 11.14
FFM (kg)		50.08 ± 12.45
Albumin (g/dL)		3.44 ± 0.59
Triglycerides (mg/dL)		119.8 ± 64.0
Total cholesterol (mg/dL)		172.1 ± 46.2
HDL (mg/dL)		54.66 ± 16.9
LDL (mg/dL)		91.80 ± 36.79
Creatinine (mg/dL)		1.19 ± 0.53
Hemoglobin (g/dL)		12.48 ± 1.93
CRP (mg/L) ^†^		4.0 (1.60–17.43)
TNF-α (pg/mL) ^†^		3.92 (3.22–5.10)
IL-6 (pg/mL)		5.52 (3.21–8.69)
Irisin (µg/mL) ^†^		7.12 (5.70–9.99)
Systolic blood pressure (mmHg)		134 ± 22.0
Diastolic blood pressure (mmHg)		76 ± 11.0
EF%		45 ± 11.0
NT-proBNP (pg/mL) ^†^		2332 (1002–4010)
LVESd (cm)		4.04 ± 0.83
LVEDd (cm)		5.45 ± 0.79
LAD (cm)		4.45 ± 0.63
RVOT (cm)		3.33 ± 0.37
TAPSE (cm)		1.91 ± 0.40
PASP (mmHg)		41 ± 11.0
NYHA	I	13 (19.7%)
	II	21 (31.8%)
	III	21 (31.8%)
	IV	11 (16.7%)
SGA	A	35 (53%)
	B	23 (34.8%)
	C	8 (12.2%)
Diabetes mellitus		28 (42.4%)
Renal failure		25 (37.9%)
ACEi treatment		40 (60.6%)
ARBs treatment		11 (16.7%)
HRT treatment		8 (12.1%)
SmokingStatus	smoker	36 (54.5%)
	non-smoker	30 (45.5%)
Cm (nF) ^†^		1.159 (0.750–1.681)
Pa (^o^)		4.09 ± 1.22
Z200/Z5		0.853 (0.830–0.879)

**Table 2:** Differences in anthropometric, metabolic, inflammatory, nutritional and cardiac parameters between chronic heart failure (CHF) patients with either presence or absence of cachexia (ACEi—Angiotensin-converting-enzyme inhibitors; ARBs—angiotensin receptor blockers; BMI—body mass index; Cm—capacitance of membrane; EF—ejection fraction; FM—fat mass; FFM—fat-free mass; HRT—Hormone replacement therapy; LAD—left anterior descending artery; LVEDd—left ventricular end-diastolic diameter; LVESd—left ventricular end-systolic diameter; NYHA—New York Heart Association; Pa—phase angle (50kHz); PASP—pulmonary artery systolic pressure; RVOT—right ventricular outflow tract; SGA—subjective global assessment; TAPSE—tricuspid annular piane systolic excursion) ^†^—non-parametric Mann-Whitney *U*-test; the other parameters were tested by parametric student’s *t*-test.

Factor		Cachectic (*n* = 34)	Non-Cachectic (*n* = 32)	*p*
Age (years)		80 ± 12	77 ± 9	0.267
Weight (kg)		71 ± 17	82 ± 17	0.010
BMI (kg/m^2^)		28.02 ± 6.22	31.64 ± 6.41	0.024
FM (kg)		23.14 ± 9.32	29.92 ± 10.89	0.020
FFM (kg)		46.61 ± 13.21	53.96 ± 10.48	0.031
Albumin (g/dL)		3.14 ± 0.61	3.76 ± 0.36	<0.001
Triglycerides (mg/dL)		118.0 ± 60.6	121.9 ± 68.6	0.806
Total cholesterol (mg/dL)		167.6 ± 52.0	177.0 ± 39.0	0.417
HDL (mg/dL)		49.90 ± 16.72	59.88 ± 15.94	0.017
LDL (mg/dL)		92.26 ± 39.88	91.28 ± 33.72	0.915
Hemoglobin (g/dL)		11.97 ± 1.84	13.04 ± 1.89	0.025
CRP (mg/L) ^†^		10.95 (1.96–26.20)	3.10 (1.45–4.95)	0.005
TNF-α (pg/mL) ^†^		4.48 (3.49–5.13)	3.29 (3.07–4.91)	0.032
Irisin (µg/mL) ^†^		7.12 (5.94–9.42)	7.61 (5.29–10.39)	0.022
Systolic blood pressure (mmHg)		132 ± 22	137 ± 22	0.409
Diastolic blood pressure (mmHg)		75 ± 12	76 ± 11	0.754
EF%		42 ± 13.0	48 ± 9.0	0.039
NT-proBNP (pg/mL) ^†^		3476 (1690–5773)	1176 (716–2605)	<0.001
LVESd (cm)		4.11 ± 0.89	3.97 ± 0.80	0.514
LVEDd (cm)		4.99 ± 0.85	5.04 ± 0.71	0.833
LAD (cm)		4.54 ± 0.72	4.36 ± 0.52	0.271
RVOT (cm)		3.35 ± 0.41	3.32 ± 0.32	0.704
TAPSE (cm)		1.84 ± 0.42	1.96 ± 0.37	0.180
PASP (mmHg)		42.8 ± 12.5	39.2 ± 10.0	0.208
NYHA I+II		13 (38.2%)	21 (65.6%)	0.030
NYHA III+IV		21 (61.8%)	11 (34.4%)	
ACEi	Yes	18 (52.9%)	22 (68.8%)	0.216
	No	16 (47.1%)	10 (31.2%)	
ARBs	Yes	8 (23.5%)	3 (9.4%)	0.188
	No	26 (76.5%)	29 (90.6%)	
HRT	Yes	1 (3%)	7 (21.9%)	0.025
	No	33 (97%)	25 (78.1%)	
SGA-A		9 (26.5%)	26 (81.3%)	<0.001
SGA-B+C		25 (73.5%)	6 (18.7%)	
Cm (nF) ^†^		0.860(0.670–1.070)	1.280 (0.916–1.789)	<0.001
Pa (^o^)		3.60 ± 1.17	4.60 ± 1.08	0.005
Z200/Z5		0.877(0.840–0.887)	0.845 (0.823–0.854)	0.002

**Table 3:** Result of the correlation study—summary of the parameters significantly correlated with plasma irisin level (Cm—capacitance of membrane; EF—ejection fraction; FM—fat mass; LVEDd—left ventricular end-diastolic diameter; LVESd—left ventricular end-systolic diameter).

Factor	R [95%CI]	*p*
**Positive Correlation**		
FM	0.408 [0.155 to 0.681]	0.020
HDL	0.318 [0.080 to 0.524]	0.010
EF%	0.253 [0.010 to 0.471]	0.046
**Negative Correlation**		
Cm	−0.393 [−0.580 to −0.167]	0.005
CRP	−0.362 [−0.561 to −0.123]	0.004
LVESd	−0.326 [−0.529 to −0.090]	0.009
NT-proBNP	−0.320 [−0.532 to −0.080]	0.010
LVEDd	−0.272 [−0.485 to −0.027]	0.030
TNF-α	−0.243 [−0.436 to −0.005]	0.044

**Table 4:** Factors selected by uni- and multivariate logistic regression analysis, that significantly affected cachexia in CHF female patients.

**Univariate Analysis**		
**Factor**	**OR [95%CI]**	***p***
Albumin	33.18 [5.00–220.4]	<0.001
Cm	10.76 [2.586–44.78]	<0.001
CRP	1.10 [1.030–1.700]	0.007
FFM	1.05 [1.0–1.111]	0.040
Hemoglobin	1.328 [1.001–1.751]	0.030
BMI	1.11 [1.01–1.210]	0.025
Body weight	1.04 [1.008–1.078]	0.013
**Multivariate Analysis**		
**Factor**	**OR [95%CI]**	***p***
Albumin	50.48 [2.594–182.39]	0.009
Cm	34.49 [4.181–282.54]	0.007
Irisin	1.390 [1.07–1.079]	0.013
Overall model fit *p* < 0.001		

**Table 5:** Tests accuracy of CRP, irisin, albumin, Cm and their combination to distinguish cachectic CHF patients from non-cachectic CHF individuals.

Factor	Sensitivity	Specificity	AUC [95%CI]	Cut-Off Value	*p*
Albumin	93.5%	52.8%	0.724 [0.582–0.788]	3.30	<0.001
CRP	55.9%	87.1%	0.704 [0.577–0.810]	9.65	0.003
Cm	52.9%	90.6%	0.787 [0.669–0.878]	0.789	<0.001
Irisin	64.7%	46.9%	0.580 [0.452–0.700]	8.11	0.270
Cm+CRP	70.6%	83.9%	0.855 [0.745–0.930]	-	<0.001
Cm+albumin	96.8%	76.5%	0.917 [0.821–0.971]	-	<0.001
Cm+Irisin	61.8%	96.9%	0.849 [0.740–0.925]	-	<0.001
CRP+albumin	96.7%	70.6%	0.858 [0.748–0.933]	-	<0.001
CRP+Irisin	58.8%	90.3%	0.731 [0.607–0.834]	-	<0.001
Cm+albumin+CRP	93.3%	85.3%	0.929 [0.938–0.979]	-	<0.001
4 markers	80%	97.1%	0.949 [0.863–0.988]	-	<0.001
